# Public Health Issue of Indoor Dilution Ventilation for Disease Prevention Versus PM2.5 in Intake Air in Auditoriums of Premier Engineering Institutes in India

**DOI:** 10.7759/cureus.25258

**Published:** 2022-05-23

**Authors:** Raja Singh

**Affiliations:** 1 Architecture, School of Planning and Architecture, New Delhi, IND; 2 ISAC Centre for Built Environment Policy, Information Sharing and Analysis Center, ISAC, National Capital Region, IND

**Keywords:** air conditioners, public health research, higher education institutes, india, indoor air quality, auditoriums, filtration, particulate matter, indoor air pollution, airborne infection control

## Abstract

Background: Dilution ventilation by enhancing fresh air intake has been prescribed to reduce airborne infection spread during the COVID-19 pandemic. This is all the more important in assembly spaces like auditoriums. Premier technology institutes have large campuses with large auditoriums for academic and cultural events in India. These institutes serve as role models for society, where gatherings are essential, but there is also the possibility of transmission of all airborne respiratory infections, including tuberculosis, into the community. The fresh air taken in should also be filtered for pollution to prevent other lung issues.

Aims: Fresh air intake and filtration have been studied in order to understand whether the outside air supplied indoors is filtered for PM2.5, which is a major ambient polluter in India.

Settings and design/methods: In this study, the Right to Information Act of 2005 has been used to obtain first-hand information from the institutes with respect to the heating, ventilation, and air conditioning (HVAC) systems in their auditoriums. Twelve of the 19 institutes fall in cities with non-attainment of ambient air quality standards.

Results: Eleven out of all those had recently integrated fresh air supply, and six replied in the negative. Only one out of all of them had appropriate filters.

Conclusion: This study highlights the need for a possible trade-off between the use of air conditioners for thermal comfort + assumed protection against PM2.5, which is the switching off of air conditioners and manually opening up windows and using fans for ventilation. Indian HVAC design for gathering spaces, especially educational institutes, needs to factor in fresh air for dilution ventilation as well as PM2.5 filtration.

## Introduction

It has been established that dilution ventilation plays a role in diluting the concentration of aerosolised droplets containing possible pathogens. Spaces where there is an ample supply of outdoor air to replace the inside air with a certain rapidity have a lower risk of airborne infection spread. The most common method of dilution ventilation is the opening of doors and windows, wherever this option is available [1-4]. This has certain downsides, like noise from outdoors, entry of insects if done without wire mesh, and a lack of comfort from air conditioning. But the major concern that is identified with the opening of doors and windows is the entry of outside dust and pollution into the interiors in urban areas. This is especially true for urban areas where the ambient air quality is poor or severe. In major Indian cities like Delhi, the major cause of poor ambient air quality is the presence of particulate matter PM2.5 [5]. The 2.5 comes from the size of the dust particles, which is below 2.5 µm. Another way of providing dilution ventilation in the space is by having fresh air in appropriate quantities mixed with the return air in an air handling unit of the heating, ventilation, and air conditioning system of the building space. This dilution ventilation is not possible in rooms with split air-conditioner systems without the opening up of windows or without placing a treated fresh air (TFA) unit if the split air-conditioner is ducted [6,7].

Using an air conditioning system without the provision of appropriate fresh air intake and constant air replacement will increase the risk of infection transmission in the indoor space [6,8]. This choice of increasing the risk of airborne infection is sometimes made with the assumption that the air conditioning system will at least filter the particulate matter present in the ambient air. This assumption is based on the guess that the air conditioner will have the capability of filtering pollution particles that are 2.5 in size (in the form of PM2.5). A trade-off needs to be made between the use of an air conditioner for comfort and the assumed protection against PM2.5, and the switching off of air conditioners and manually opening up windows and using fans for ventilation. The worst situation is when the air conditioner neither provides appropriate fresh air for dilution ventilation (and airborne disease reduction) nor filters the harmful PM2.5 particles, which are harmful to human lungs.

In this study, we compiled the information on heating, ventilation, and air conditioning (HVAC) systems of India’s premier engineering institutes, which set the benchmark for state-of-the-art research in India. The study showed that even though many of the systems complied with providing fresh air (though not always quantified), most had filters that could not filter the particulate matter PM2.5. The study may be a benchmark for the situation of air conditioning systems in other buildings. In India, neither are airborne infections new, as we have yearly cases of tuberculosis, nor is the harmful effect of PM 2.5 unknown [9]. In such a scenario, our air conditioning systems should incorporate appropriate fresh air changes and appropriate microfilters for healthy indoor air quality.

Literature background

In response to the COVID-19 pandemic, major HVAC institutions such as the American Society of Heating, Refrigeration, and Air-Conditioning Engineers (ASHRAE) from the United States, the federation of European Heating, Ventilation, and Air Conditioning Associations (REHVA) from the European Union, the Society of Heating, Ventilation, and Sanitary Engineering from Japan, major institutional bodies from China, the Indian Society of Heating, Ventilation, and Air Conditioning Engineers and the Central Public Works Department of India have all released documents for the operation of air conditioners in buildings. They all agreed that intake of outdoor air with appropriate patterns of airflow is one of the most important strategies to prevent or reduce transmission of the COVID-19 virus [3,10-15]. This intervention has been advocated by practitioners of tuberculosis prevention and control [2-4,16-19]. But it is also true that the outdoor air in cities with high PM2.5 pollution enters the indoors and causes lung issues for inhabitants [20-22]. This is all the more severe in Indian cities, which have high urban outdoor pollution, especially suspended particulate matter [5,23]. In some cases of enclosed spaces, the indoor concentration of PM2.5 was much greater than that of the outdoor air [24,25]. HVAC systems must not be designed with only one parameter in isolation. The airborne infection control measure of using dilution ventilation must take care of the PM2.5 pollution as well as dilution ventilation for airborne infection control. The filtration must have a minimum efficiency reporting value (MERV) capacity of ≥10 as it will provide a lower concentration of PM2.5 indoors [26]. Other factors like energy consumption must also be taken into consideration [21]. An integrated approach is required at the design stage [12,27-29]. The author has conducted another study along the same lines with other important Indian buildings [30]. This study has also been done for Indian airport buildings [31].

Question: Do the air conditioning systems of auditoriums of premier engineering institutes have appropriate provisions for dilution ventilation and appropriate filtration capability to filter out particulate matter PM2.5?

Need for the study

Large assemblies in educational institutes or elsewhere happen in auditoriums. These assembly spaces are very frequently air-conditioned. During the COVID-19 pandemic, we saw recommendations for increasing the fresh air intake into enclosed spaces by opening the windows or by fresh air intake through the air handling unit of the HVAC system. These HVAC systems must be equipped to enable fresh air intake as this dilutes the concentration of aerosolised pathogens and decreases the risk of airborne infection spread. But in-letting of outside air has also raised concerns about in-letting pollution, especially in India and China due to particulate matter, especially PM2.5. This study focused on premier engineering institutes, which are at the zenith of research and serve as role models for society. There is a specific focus to understand whether the filtration systems are equipped to filter the PM2.5 when we want to increase the fresh air intake for the reason of airborne infection prevention.

Practical implications of the study

This study highlights the Indian reality where HVAC design needs fresh air intake as well as PM2.5 filtration to prevent airborne infections and particulate lung diseases, respectively. This study also uses a unique data collection method where the Right to Information Act of 2005 has been used to get authentic information from the first-hand managers of the space.

## Materials and methods

The stepwise methodology used in this study has been presented in Table 1. 

**Table 1:** Methodology used in this study.

S. No.	Step
1	Preparation of a focused set of questions for procuring related data.
2	Obtaining information using the Right to Information Act, 2005.
3	Compiling information and standardising it for comparison.
4	Discussion of results and reporting information.
5	Recommendations.

Selecting premier engineering educational institutes

In this study, two important sets of premier institutes imparting engineering education were selected. This includes the Indian Institutes of Technology and the National Institutes of Technology. There are 23 Indian Institutes of Technology and 29 National Institutes of Technology [32,33]. This makes a total of 52 premier, top-run engineering institutes in India. In late 2020, before the second wave in many parts of India, the government had started opening up premier institutes for certain sections of students and most of the faculty members. There were cases in major premier institutes of rising cases, and some of them became hotspots. These premier institutes are directly managed by the Central Government and are well funded. They also hold a major chunk of research in India. These institutes are also spread evenly across the whole country. All of the institutes have all the facilities for students, including assembly spaces for the large events that happen in these institutes. These are also the most well-built, and most are assisted by the faculty of the institute itself. All of these have auditoriums with a very large capacity for important fests, convocations, annual days, etc., which are important events for a college. Being educational institutes, they would also respond well to research-based studies.

Information was derived from the public information officers of the educational institutes. The information was sought under the Right to Information Act, 2005. This enables information that is in the public domain. The information was asked in the format mentioned in Table 2.

**Table 2:** The information sought under the Right to Information Act, 2005 under the public domain for the premier engineering institutes. HVAC: heating, ventilation and air conditioners, UVGI: ultra violet germicidal irradiation, HEPA: high efficiency particulate air filter, COVID-19: Coronavirus disease of 2019, SARS: severe acute respiratory syndrome.

S. No.	The Information Sought under the Right to Information Act, 2005 under the Public Domain.
1	Provide the details of the Air Changes per Hour inside the various spaces within the auditorium. (Air changes per hour is a unit to measure the amount of fresh air that will be supplied to the auditorium for dilution ventilation inside the built space.)
2	Provide the details of the HVAC that has been put in the auditorium along with its ventilation details, i.e., the technical specifications for the provision of fresh air and the exhaust of the interior air into and from the inside spaces, respectively.
3	Is there a provision of UVGI treatment of the air which is to be supplied to the inside of the auditorium.
4	Please specify the details of the filters (HEPA or others) that will be provided in the HVAC system of the auditorium. Also mention the least count of the filters which will tell us the minimum size of particles (dust or microbes) it can filter.
5	What additional measures have been integrated in the design of the building to prevent the spread of airborne disease like COVID-19, Tuberculosis, Measles, SARS etc.?
6	Is there any interaction of real-time monitoring with meters/sensors of the levels of carbon dioxide in the ventilation/HVAC system of the auditorium?
7	What is the capacity of the auditorium in the peak time?

The information presented in Table 2 was requested in the form of an Application of Information under Section 6 of the Right to Information Act, 2005, which empowers any citizen to obtain any government record in the country held by public authorities [34]. This is a boon for researchers as they may have to face bureaucratic hurdles in obtaining information. This study uses this tool to derive information from the premier government-run institutes, and the data have been satisfactorily provided. The above-mentioned information requests were drafted into applications which were sent in the format of applications according to the Right to Information Act, 2005 [34,35]. The rationale, or the intent of the information requested, has been provided in Table 3.

**Table 3:** The intent of the specific information requested from the premier engineering institutes. HVAC: heating, ventilation and air conditioning, UVGI: ultra violet germicidal irradiation.

S. No.	Intent
1	Fresh air intake through the HVAC system. This is because fresh air intake aids dilution ventilation, which is key in the lowering of concentration of aerosolised pathogens.
2	The presence of UVGI system in the HVAC. This is of key importance as UVGI systems neutralise the pathogens that may be aerosolised in the space, or the one which passes through the ventilation mechanism [36].
3	The filtration and its details in the HVAC system. The filtration serves a double purpose. One is a possible filtration of the pathogens or their aerosols and the filtrations of other pollutants like dust and suspended particulate matter.
4	Real-time monitoring of the mechanism of ventilation. This is key as it will automate the process and the air intake can be increased based on the bio response from the number of people in the space who would exhale carbon dioxide and a certain increase would possibly trigger a response for more inflow of outside air. In this case, the carbon dioxide serves as a biomarker or a surrogate for the outside air ventilation into the enclosed space [27].

The replies to the applications were received by the author over a period of time. Some applications had to be followed up on, and appeals on some others had to be filed. The resultant was a reply from 19 premier institutes that gave an authorised reply to the questions, which has been the basis of this study. The various institutes and their in-house engineering and estate management teams responded to the applications as per their understanding. There were also variations in the units of measurement in the replies. Diversity was also present in the way in which the reply was made. This study took the applications and standardised them in common units and common points were made in order to compare and compile the data easily.

Sample size justification of the study

The total number of top-run, premier, and public-funded engineering institutes in India is 52. This includes 23 Indian Institutes of Technology and 29 National Institutes of Technology. The number of institutes under the study is 19. This means that, using the Survey Monkey sample size calculator, the confidence level is 90% with a margin of error of 15.5%. The margin of error may be more as there may be issues with the reporting of the information and/or any errors. The period of the data collection for the study was late 2020 to early 2021. The systems in the auditoriums of these auditoriums may have been changed after that period. The changes have not been included in this study.

Exemption from ethics review committee

The paper has no human participants or living human or animal tissue. All the information is available in the public domain. This is because the Right to Information Act of 2005 enables the provision of information in the public domain. The information under the Act is also free from any third party's personal information as an essential component of the act. This means that the paper is exempt from the Ethics Review or Institutional Review Board in line with the Indian Council of Medical Research: National Guidelines for Biomedical and Health Research Involving Human Participants [37]. The author declares the same.

## Results

The data received from the 19 premier engineering institutes have been compiled and presented in Appendices Table 6.

On compilation and comparison, it was found that: first, 12 out of the 19 responding institutes were on the list of ‘Non-Attainment of Air Quality Standards’ [38] released under the National Clean Air Program [5]. The list was prepared by the Central Pollution Control Board. The presence in the list means that the city of the institute has air quality below the standard and there are a higher number of days when the particulate matter, especially PM2.5 would be above the permissible limits. Second, out of the 19 institutes that responded about their auditoriums, six replied to having air changes per hour as per the National Building Code of India 2016, which stipulates air changes per hour to be 4-8 in assembly spaces [27]. Five of these institutes responded with values that were below the NBC stipulated value. Six did not provide a reply or the reply was unclear, and two of the institutes did not have air conditioning or any form of HVAC system except basic fans and windows (and exhaust fans). Third, out of the 19 responding institutes, 11 stated that they have had or have recently increased or provided fresh air intake into the HVAC system in the auditorium space in their institute. Six responded in the negative about the fresh air intake into the auditorium space. The remaining two were not applicable as they had naturally ventilated auditorium spaces, with room fans, windows, etc. Fourth, on the issue of carbon dioxide monitoring, out of the 19 institutes which responded about their auditoriums, two had carbon dioxide monitoring integrated into their HVAC system, which also created a feedback mechanism for the air supply into the AHUs. Fifteen institutes responded in the negative, and two provided unclear answers. Fifth, on the question of the provision of UVGI in the auditorium space or integrated within the HVAC system, only one auditorium responded positively. Sixth, no college out of the 17 eligible institutes had HEPA filters in the HVAC systems of their auditoriums. Two institutes out of the 19 were naturally ventilated.

The filtration system in the HVAC systems of the auditoriums had different grades of filters. The filter system of the HVAC for the auditoriums in the various institutes is given in Table 4.

**Table 4:** The types of filters used in the auditoriums of the institutes along with some detail. MERV: minimum efficiency reporting value, HVAC: heating, ventilation and air conditioning.

S. No.	No of auditoriums	Filter grade
1	1	MERV 13 (fine dust filters having certain higher efficiency in filtering particles from 0.4 microns to 10 microns) [39]
2	12	MERV 6-8 (or simply thick dust filters having very low efficiency in filtering fine dust particles in the aerodynamic diameter from 04 microns to 10 microns)
3	2	MERV 5-6 (lower efficiency as compared to MERV 8)
4	2	Original equipment manufacturers built in filters. Their results are not widely tested and published. These are generally placed on the split, cassette, and other standalone air conditioners.
5	2	N.A. No filters as the auditoriums had no HVAC system.

The least count of the filter as defined by the in-house engineering teams of the various institutes in which the auditoriums are located and whose HVAC systems are being studied is given in Table 5.

**Table 5:** The compilation of the least counts of filters provided by the institutes.

S. No.	No. of auditoriums	Least count of filter in microns
1	5	10 micron
2	1	3 micron
3	1	5 micron
4	2	Not applicable as there was no HVAC in these auditoriums.
5	9	Data not provided/not clear
6	1	2.5 micron in the form of original equipment manufacturer supplied internal filter.

## Discussion

First of all, it is important to understand that most institutes made extra arrangements in response to the pandemic COVID-19 and that the increase in air changes was a quick-fix response to the pandemic. This is positive as it exhibits an eager response, but it is negative as it ignores the presence of existing airborne threats that have been in India for a long time. This includes tuberculosis, which has been an active killer in India [4,9,40]. Having appropriate measures in enclosed spaces in India should have been a standard procedure as the disease is in the community in India.

The second point of discussion is the basis of the study in this paper, which explores the idea of insufficient filtration of particulate matter, especially PM2.5, by air conditioning systems, making these air-conditioned spaces neither fully safe from pollution nor against the aerosolized suspended pathogens. This is of major concern as the opening of windows, though necessary for dilution ventilation, is discouraged in certain cities as there is a perceived, and maybe an actual, concern about particulate matter that may enter the buildings. A common response to this is to switch on the air conditioner and seal the windows of the room. This is of concern as this may lead to lower fresh air changes inside of the room and therefore an increased risk of airborne disease spread. There is a certain population that may switch on portable air filters, but this has been discouraged by the World Health Organisation [41]. Another important point is how India is uniquely placed in this situation and has 132 cities with non-attainment of the prescribed ambient air quality measures, with particulate matter being the biggest threat [38]. The criteria for inclusion in the non-attainment list of studies included five-year monitoring for ambient air, where if there is an excess of suspended particulate matter (or nitrogen dioxide) as per the National Ambient Air Quality Standards [31,42,43]. Of our list of 19 auditoriums, 12 were located in cities that were part of the non-attainment list. Any design of HVAC for assembly spaces in India should definitely factor in the need for filtration of particulate matter, which may not be the case for standard designs in some countries with cleaner air. Academic institutions have huge populations of students who gather in their auditoriums. Auditoriums in general are supposed to be the model for sound ventilation. This is because there is always a specialised design for these and they can become epicentres for large-scale airborne disease spread events. Having appropriate ventilation and good indoor air quality is a public health issue and not just an engineering problem [28]. This is a right that has been included in the right to a healthy environment and forms part of the Right to Life [44], a fundamental right guaranteed by the Indian Constitution [45]. The COVID-19 pandemic has exposed the need for stricter action against the indoor air crisis that we are facing [46].

## Conclusions

Eleven out of all the institutes whose auditorium air conditioning was studied had recently integrated fresh air supply and six replied in the negative. Only one out of all had the appropriate filter required to supply filtered fresh air for proper dilution ventilation, leading to possible airborne infection control. This one was in a city not meeting the non-attainment criteria for outdoor pollution. Most others who lived in cities with ambient pollution, especially particulate matter, did not have filters to filter out the particulate matter with a diameter of 2.5 microns. It is therefore highly recommended that for large assembly buildings there should be appropriate measures that not only let in fresh air but also filter the outside air appropriately in order to exclude particulate matter. These buildings, especially when located in premier institutes, should serve as model buildings for other assembly spaces to follow. This may be achieved by the inclusion of HEPA filters or filters with MERV ≥10 and above, along with a generous supply of outside fresh air. This may increase the energy cost as the outside air has to be constantly conditioned and there is a reduction in recirculated air. This step may also automatically stop the usage of recirculated air retaining mechanisms in the HVAC systems of the buildings. An increased energy cost in return for the safety and well-being that users of the space will get is their right. There should be proper preventive maintenance, a record of fresh air provisions, and their attainment should be set on record so that continuous compliance is assured. Committees for enforceable standards must be constituted. Every building must display to the users, prominently, the current status of breathable air, its quality, and the mechanisms to ensure their well-being in the space.

## Appendices

**Table 6:** The compilation of the data received from the 19 premier technology institutes of India. These data have been derived from the replies to the request for information provided under the Right to Information Act, 2005. The data are in the public domain. The data have been interpreted in the best possible manner in good faith. Any discrepancies may be reported, and will be corrected at the earliest. The period of the data collection is late 2020 and early 2021. The changes in the systems at the institutes have not been made in this set of data. Complete data may be made available on reasonable request. IIT: Indian Institute of Technology, NIT: National Institute of Technology, BRA: B. R. Ambedkar CO_2_: carbon dioxide, NCAP: National Clean Air Programme, MERV: minimum efficiency reporting value, EU: European Union, OEM: original equipment manufacturers, AV: audio video, ACH/ACPH: air changes per hour, UVGI: ultra violet germicidal irradiation, NBC: National Building Code, SV: Sardar Vallabhai Patel, BHU: Benaras Hindu University, CFM: cubic feet per minute, LHC: Lecture Hall Complex.

S. No.	Institute	Whether NCAP non-attainment city	ACH if mentioned 4-8	ACH as per NBC 2016	Whether fresh air provision in HVAC Yes/No	Fresh air quantified	CO_2_ monitoring	UVGI	Whether HEPA filter provided	Filter provided	Filter least count/efficiency
1	A large congregational library of a premier technology institute in New Delhi (Central Library, IIT Delhi)	Yes	3 to 4	Yes	Yes	100 % fresh air, no return.	No	No	No	Pre-filter (MERV 8 or less)	Not provided
2	An auditorium of a premier technology institute in New Delhi (Dogra Hall, IIT Delhi)	Yes	4 to 6	Yes	Yes	17733 CFM fresh air.	No	No	No	Pre-filter (MERV 8 or less)	Not provided
3	The largest auditorium of an premier technology institute in North India (Convocation Hall, IIT Roorkee)	No	12 (in case of fire)	Yes	Yes	17733 CFM fresh air.	No	Yes	No	MERV 13 EU 7	3 micron/not provided
4	A large lecture hall located in the lecture hall complex of a prominent technology institute in Western India (Hall 308, LHC, IIT Jodhpur)	Yes	Not provided	Not provided	No	Not provided	No	No	No	MERV 8	10 micron/90%
5	A convention hall of a large premier institute in the metro city of Mumbai (Victor Menezes Convention, IIT Mumbai)	Yes	1	No	Yes	Not provided	No	No	No	MERV 7-8 EU 4	10 micron/not provided
6	The Main Auditorium in a prominent technology institute in the Indian state of Uttar Pradesh (IIT Kanpur)	Yes	>10	Yes	Yes	100 fresh air provision	No	No	No	Not specified (MERV 8 or lesser)	10 micron/ 80%
7	An auditorium belonging to one of India’s largest premier institute located in Eastern India. (Netaji Auditorium, IIT Kharagpur)	No		Not provided	No	Not provided	No	No	No	Pre-filter (MERV 8 or less)	Not provided
8	Another auditorium belonging to the Eastern Indian premier institute (Kalidas Auditorium, IIT Kharagpur)	No		Not provided	No	Not provided	No	No	No	Pre-filter (MERV 8 or less)	Not provided
9	The main auditorium located in the academic block of a premier institute located in central India, IIT Hyderabad Hyderabad	Yes	9 ACPH 2.42 Fresh ACPH	yes	Yes	2500 CFM fresh air 2.42 fresh ACPH	No	No	No	MERV 5-6 EU 3	Not provided
10	Main conference room located in the auditorium building of a prominent North East Indian institute (IIT Guwahati)	Yes	1	No	No	Not clear	No	No	No	Pre-filter (MERV 8 or less)	Not provided/30%
11	Auditorium of a premier institute in Western India (Jesubhai Memorial Auditorium, IIT Gandhinagar)	Yes	1	No	Yes	Fresh air 1 ACPH	Yes	No	No	Pre-filter (MERV 8 or less)	10 micron
12	Auditorium of a premier institute in Northern India (Prof. Gopal Tripathi Auditorium, IIT BHU)	Yes	Naturally ventilated	N.A.	N.A	Not applicable	No	No	No	N.A.	Not applicable
13	Auditorium in a Student Activity Centre of a premier technology institute of South India (IIT Madras)	No	1.5	No	Yes	Not provided	Yes	No	No	Not provided (MERV 5-6 or lesser)	Not provided
14	A seminar hall, located in a premier institute in Western India (Production Seminar Hall, SV-NIT Surat)	Yes	<1 (because of split ac)	No	No	Not clear	No	No	No	OEM filters	2.5 micron OEM/not provided
15	An audio visual auditorium located in a premier institute in Central India (Bhubaneshwar Behera AV Room, NIT Rourkela)	No		Not provided	Yes	15% of design condition fresh air	No	No	No	Pre-filter (MERV 8 or less)	5 micron/not provided
16	An auditorium in a South Indian premier institute (Dr. BR Ambedkar Learning Centre, NIT Warangal)	No	12	Yes	No	Not clear	No	No	No	OEM filters	Not provided
17	An auditorium in a central Indian premier institute (VNIT Nagpur)	Yes		Not provided	Yes	9800 cfm fresh air	Not clear	No	No	Pre-filter (MERV 8 or less)	10 micron/90%
18	The main auditorium of a premier institute in Southern India (NIT Calicut)	No	Naturally ventilated	N.A.	N.A.	Not applicable	Not clear	No	No	N.A.	N.A.
19	The central seminar hall located in a premier institute in Northern India (Central Seminar Hall, BRA-NIT, Jalandhar)	Yes	Not provided	Not provided	Yes	Return duct closed. 100% fresh air.	No	No	No	MERV 7-8 EU 4	Not provided
